# Understanding and measuring workplace violence in healthcare: a Canadian systematic framework to address a global healthcare phenomenon

**DOI:** 10.1186/s12873-024-01144-1

**Published:** 2025-01-13

**Authors:** Christian Schulz-Quach, Brendan Lyver, Charlene Reynolds, Trevor Hanagan, Jennifer Haines, John Shannon, Laura Danielle Pozzobon, Yasemin Sarraf, Sam Sabbah, Sahand Ensafi, Natasha Bloomberg, Jaswanth Gorla, Brendan Singh, Lucas B. Chartier, Marnie Escaf, Diana Elder, Marc Toppings, Brian Hodges, Rickinder Sethi

**Affiliations:** 1 Temerty Faculty of Medicine, University of Toronto, Toronto, Canada; 2 University Health Network, 200 Elizabeth St, Toronto, ON M5G 2C4 Canada; 3 Lawrence S. Bloomberg Faculty of Nursing, University of Toronto, Toronto, ON Canada; 4 Department of Family Medicine, McMaster University, Hamilton, ON Canada

**Keywords:** Workplace Violence in Health Care, Quality Improvement, Emergency Department, Pandemic Recovery, Health Systems Innovation

## Abstract

**Background:**

Globally, healthcare institutions have seen a marked rise in workplace violence (WPV), especially since the Covid-19 pandemic began, affecting primarily acute care and emergency departments (EDs). At the University Health Network (UHN) in Toronto, Canada, WPV incidents in EDs jumped 169% from 0.43 to 1.15 events per 1000 visits (*p* < 0.0001). In response, UHN launched a comprehensive, systems-based quality improvement (QI) project to ameliorate WPV. This study details the development of the project’s design and key takeaways, with a focus on presenting trauma-informed strategies for addressing WPV in healthcare through the lens of health systems innovation.

**Methods:**

Our multi-intervention QI initiative was guided by the Systems Engineering Initiative for Patient Safety (SEIPS) 3.0 framework. We utilized the SEIPS 101 tools to aid in crafting each QI intervention.

**Results:**

Using the SEIPS 3.0 framework and SEIPS 101 tools, we gained a comprehensive understanding of organizational processes, patient experiences, and the needs of HCPs and patient-facing staff at UHN. This information allowed us to identify areas for improvement and develop a large-scale QI initiative comprising 12 distinct subprojects to address WPV at UHN.

**Conclusions:**

Our QI team successfully developed a comprehensive QI project tailored to our organization’s needs. To support healthcare institutions in addressing WPV, we created a 12-step framework designed to assist in developing a systemic QI approach tailored to their unique requirements. This framework offers actionable strategies for addressing WPV in healthcare settings, derived from the successes and challenges encountered during our QI project. By applying a systems-based approach that incorporates trauma-informed strategies and fosters a culture of mutual respect, institutions can develop strategies to minimize WPV and promote a safer work environment for patients, families, staff, and HCPs.

**Supplementary Information:**

The online version contains supplementary material available at 10.1186/s12873-024-01144-1.

## Background

### Problem description

Workplace violence (WPV) in hospitals is a multifaceted problem that impacts healthcare institutions globally [1, 2]. The definition of WPV in the healthcare setting is heterogeneous across different agencies and policy makers (see Table 1). However, following the onset of the COVID-19 pandemic, the rate of WPV has more than doubled in healthcare institutions around the world, especially in high-acuity and emergency department (ED) settings [3, 4]. Although there exist numerous attempts by hospitals to address WPV through various interventions, individual initiatives have encountered challenges in achieving a positive impact in part due to the complexity of WPV [5].

**Table 1:** Comparing Workplace Violence and Code Whites, [2]

**Definition of Workplace Violence (World Health Organization)**	**Definition of Workplace Violence (Occupational Health and Safety Act,. R.S.O, 1990, c. O.1)**	**Definition of Code White (Public Services Health & Safety Association, Ontario, Canada)**	**Definition of Code White (University Health Network, Toronto, Canada)**
**Global Definition**	**Provincial Definition**	**Provincial Definition**	**Local Definition**
" … incidents where staff are abused, threatened or assaulted in circumstances related to their work, including commuting to and from work, involving an explicit or implicit challenge to their safety, well-being or health." (Richards, 2003, p. 2)	a. The exercise of physical force by a person against a worker, in a workplace, that causes or could cause physical injury to the worker; b. An attempt to exercise physical force against a worker, in a workplace, that could cause physical injury to the worker; c. A statement or behaviour that it is reasonable for a worker to interpret as a threat to exercise physical force against the worker, in a workplace, that could cause physical injury to the worker.	**Definition:** Code White is a coordinated and trained emergency response to a care recipient, worker, or visitor displaying violent behaviours that may cause harm or injury to others, themselves, and/or is damaging to property. **Reasons:** Worker perceives themselves or others in danger from a person’s behaviours; Person’s behaviours are harmful to self, others, or damaging to property; Person’s behaviours are escalating towards physical violence; Person’s behaviours are unmanageable for workers and resources.	**Definition:** An emergency response for a violent person. **Reasons:** The person is verbally and/or physically threatening towards themselves, staff, patients/clients, and/or visitors; The person is not responding to verbal de-escalation techniques, negotiating, redirection, limit setting, and problem-solving techniques by the staff; The person may require restraint (chemical and/or physical) and is anticipated to be resistive to the restraining procedure; and/or, urgent assistance is required.

**Fig. 1:**
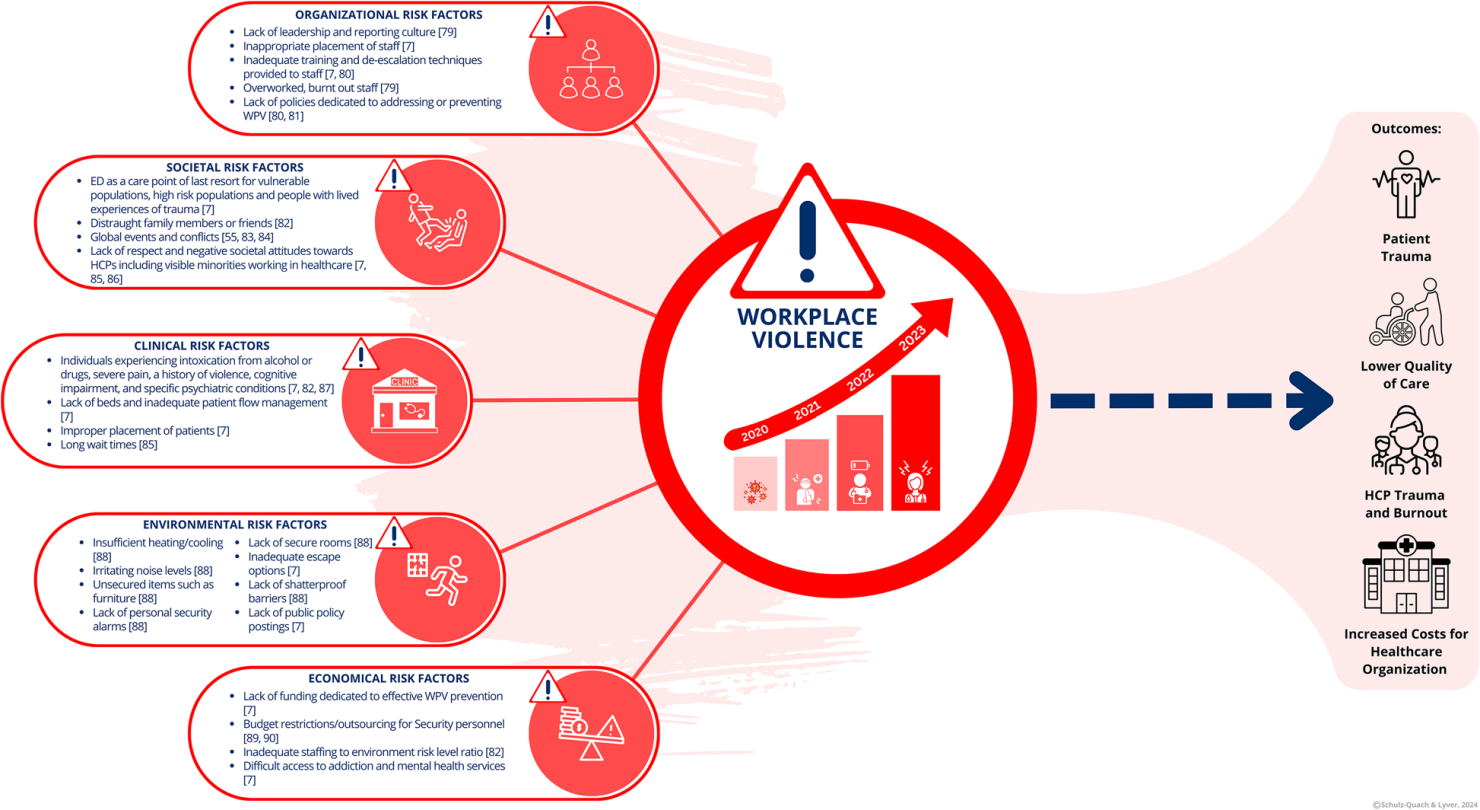
This figure illustrates the complexity of WPV in healthcare, encompassing the escalation of WPV since the onset of the pandemic, the contributory risk factors, and the resultant outcomes for patients, HCPs, and healthcare organizations. References relating to [7, 9–21]

Ameliorating WPV-related outcomes requires complex interventions and a systems approach to change management in healthcare [5, 6]. To effectively design such interventions, a comprehensive understanding of the issue and contributing risk factors is required. Several category systems for WPV in healthcare settings have been proposed, such as the five risk factor categories suggested by Keith & Brophy [7]: (1) clinical risk factors, (2) environmental risk factors, (3) organizational risk factors, (4) societal risk factors, and (5) economic risk factors [7, 8]. Figure 1 illustrates the breadth of these factors, emphasizing the interdependence of variables contributing to WPV. A systematic framework and step-by-step guidance for the development and implementation of WPV prevention interventions is needed in healthcare to support organizations in making measurable impacts on this multifaceted problem.

### Available knowledge

A multitude of theories in scholarly research provide deep and varied understanding of the factors that drive WPV in healthcare environments [22]. These theories arise from a range of academic fields, such as psychology, biology, criminology, ecology, and sociology. Table 2 provides a summary of theoretical frameworks identified in a narrative review of the literature to describe WPV. Gaining insight from various theoretical standpoints is essential for a cross-disciplinary approach to effective WPV prevention strategies in healthcare settings and it was one of the early steps our team approached when initiating this project [22]. We believe that the practical application of these theories is key to understanding the subtle aspects relevant to intervention(s) implementation, which can greatly influence their success. For example, the efficacy of common practices such as education and training programs, commonly used in WPV prevention strategies, may hinge on how well these theories are integrated into their design. Theories such as the Minority Stress Model [23], Terror Management Theory [44], and the Struggle for Recognition theory [30], highlight the importance of adopting a trauma-informed perspective which has been linked to improved outcomes in healthcare settings [50, 51]. Without an in-depth understanding of these theories, training may prove to be inefficient or yield only temporary effects without the necessary impact on organizational culture change. However, research has demonstrated that well-structured training programs can reduce the impacts of WPV incidents [52]. Thus, good understanding of WPV theories is crucial for effectively addressing and ameliorating WPV in healthcare settings.

**Table 2:** Summary of theoretical frameworks identified in our literature review for understanding WPV

Theory	Theory description	Category of risk factor	Non-modifiable factors within hospitals	Modifiable factors within hospital	Quality improvement initiatives
Ecological occupational health model of workplace assault [23, 24]	• A model depicting the factors and consequences of WPV in Long Term Care • It considers the complex interactions between individual worker, interpersonal, organizational environment, and societal factors. • Factors included the individual worker, the workplace, the external environment, and the assault situation • Assaults have an impact on the workers, workplace and quality of care	• Clinical risk factors • Societal risk factors • Organizational risk factors	• Societal changes • crime rates • substance abuse • lack of mental health care • Staffing levels • Global affairs/wars	• Individual worker sense of safety in their workspace	• Environmental indicators • Debriefing Improved • Incident Reporting • Interviews, pulse surveys
				• Training and education	• Educational intervention
Broken Windows Theory [25]	• Criminology based • Tolerance of smaller crimes leads to more larger crimes occurring • Tolerance of verbal abuse can lead to more severe forms of abuse, such as physical abuse, being tolerated	• Organizational risk factors	• Societal changes • crime rates • substance abuse • lack of mental health care • world events	• WPV Policies Managing • WPV Data and statistics	• Identifying and implementing tailored quality indicators on WPV
Cultural care theory [26]	• Uses Holistic Culture Care Theory • Identifies 4 subcultures of those involved in ED WPV • ED Nurses • Institution’s administration departments • Clients with violent behaviours • Clients without violent behaviours • Three prominent themes • Policies often lack sufficient contribution to a safe ED environment • Administration often value hospital/organizational reputation over ED staff well-being • Anxiety, fear, and negative emotions caused by WPV impact nurses’ quality of care	• Clinical risk factors • Societal risk factors • Organizational risk factors	• Clients with violent behaviours Clients without violent behaviours	• WPV culture • Support for ED staff	• Educational interventions • Environmental indicators • Debriefing • Improved Incident Reporting • Interviews, pulse surveys • Clinical representation in leadership decisions
Routine Activity Theory [27]	• Criminology based • A person’s daily activities can increase or decrease the opportunities for victimization • A person with one or more of the following is likely to be a victim of WPV; • greater exposure to aggressor • lower levels of protection over possible targets • repeated engagement with potential aggressor • greater closeness to potential aggressor	• Organizational risk factors • Societal risk factors • Clinical risk factors • Economical risk factors	• Patient behaviour upon arrival • Staff exposure to patients with aggressive behaviours	• Societal perspectives on hospital staff • Increased protection for staff	• Environmental indicators • Increased security presence • Wearable devices such as body cameras • Personal alarm buttons • Environmental awareness training
Situational Crime Prevention Theory [28]	• Criminology based • Situational crime prevention theory is based on rational choice theory • Offenders consider the perceived risks and rewards associated with crime • in healthcare, attacking a nurse could prevent the pain associated with medical interventions such as drawing blood • Need to emphasize the punishment and risk for violent behaviour in ED in environment to reduce the rewards	• Organizational risk factors		• Emphasizing WPV policies in hospital	• Environmental indicators • Policy review
Framework of cultural aspects of violence in the ED [29]	• Cultural themes related to WPV in the ED were categorized into the following three groups; • Problems and solutions • Staffs perceptions of violence • Indicators or warning signs of violence • Diverse staff responses to violence • Them and us • Patient/relative behaviour • Nurses’ behaviour • Requests and demands • Rejected request of patients/relative • Long waiting times or waiting times perceived as long contributing to requests and demands of patients and relatives	• Organizational risk factors • Societal risk factors • Clinical risk factors • Economical risk factors	• Wait times • Patient and relative behaviour • Number of staff	• Nurses behaviour • Perceptions of violence • Knowledge of violence • Response to violence • Patient/visitor requests	• Education • Support • Outreach to staff • Trauma informed approach • Behavioural emergency response team
Honneth’s theory of struggle for recognition [30, 31]	• Three themes that describe the experiences of patients in WPV situations; • Unmet needs • Involuntary assessment • Unsolicited touch • Violence felt as a demand for rights and recognition as a person	• Societal risk factors • Clinical risk factors	• Previous patient experiences and traumas	• Trauma-informed approach	• Education for staff
Psychological frameworks [32]: Psychoanalytical model [33], personality theories [34–39], frustration-aggression hypothesis [40]	• Aggressive behaviours may be innate and unconscious forces due to certain personality types • Frustration when patient can’t get what they want • We have no control over who enters ED so nurses must be calm and prepared	• Societal risk factors	• Patient population	• Calm and prepared HCPs	• Education interventions • Support for staff • Pulse survey, check ins • Behavioural emergency response team
Environmental Stimuli theories [32]	• Negative affect escape model [41] • Unpleasant environmental stimuli increasing in intensity can often lead to aggression • Excitation-transfer theory [42] • Stimuli add up and can result in triggering an aggressive behaviour	• Environmental risk factors	• Patient population with environmental triggers • Physical layout of hospital	• Limiting stimuli • Comfortability of patients and visitors	• Seclusion rooms • Limiting noise • Regulating temperature • Creating calm atmosphere
Rational Choice Theory [43]	• Makes choice based on rational calculation of costs and benefits or pain versus enjoyment with the aim of maximizing pleasure • Perception and understanding of potential pain caused by the punishment drives the choice • If you don’t follow through with protecting you workers, then likely to commit more violence as no costs, only benefits	• Clinical risk factors • Organization risk factors	• Patient population with prior negative experiences and traumas with healthcare • Patient population with knowledge of medicine	• Trauma-informed approach • Supported staff • Policy to protect staff	• Education interventions • Debriefs • Improved culture with regards to WPV • Environmental indicators • Policy change
Terror Management Theory [44]	• Addresses how individuals cope with anxiety and fear associated with their awareness of mortality • If a person consciously believes that a world event such as the • Covid-19 virus could result in death, fear of death will play a role in a person’s attitude and behaviours regarding the topic Covid-19 disrupts the feeling of calmness, causes a feeling of threat, terror management takes over and responds in distress or disorderly manners • Awareness of death creates a potential for existential terror to due survival instincts • Anxiety buffering systems related to cultural worldviews, self-esteem and close interpersonal relationships play a role in this • Fear of death may be conscious or unconscious	• Clinical risk factors • Organization risk factors	• Patient population with prior negative experiences and traumas with healthcare • Patient population with knowledge of medicine	• Trauma-informed approach • Supported staff	• Education interventions • Debriefs • Improved culture with regards to WPV • Increased security guard presence
Psychology Model for Understanding Violence [45]	• Psychobiological theories of violence include brain dysfunction, autonomic functioning, hormones, neuropsychology, and temperament • Evolutionary psychology states that due to natural selection, humans have evolved adaptations enabling them to harm other humans in order to reproduce and survive, which still exists • The death instinct states that a defence system controls our anger but when we no longer feel like they keep us safe, we may react with violent behaviour	• Societal risk factors	• Biological factors affecting patient population	• Trauma-informed approach • Culture change	• Education intervention • Environmental indicators
Sociological perspectives on WPV [46]	• Violence is a resource used to obtain a result • Violence is a reaction to a crisis situation • Culture of violence from either, friends, family, community or society leads to more violence	• Societal risk factors	• Patient population	• Trauma-informed approach	• Education intervention
Psychosocial Risk Factors [47]	• Biological vulnerability for aggression in combination with psychosocial factors contribute to aggressive behaviours, factors include • Proximal factors, present psychological state • Developmental and environmental factors influencing an individual’s personality and cognition	• Societal risk factors	• Patient population	• Agitation management	• Education Intervention
Minority Stress Model [48]	• A model based on multiple sociological and social psychological theories that describes the stigma, prejudice, and discrimination that minority communities encounter and the impact that these negative experiences have on individuals’ physical and mental health • Negative encounters stemming from discrimination and stigmatization may lead individuals to anticipate rejection and discrimination in social contexts, prompting the development of defensive coping mechanisms that could be perceived as confrontational • Marginalized communities can include ethnic minorities, the 2SLGBTQIA+ community, individuals with disabilities • Model argues that minority stress can only be managed through systemic changes that address social inequalities and providing the necessary supports and resources that marginalized communities require	• Societal risk factors	• Societal inequalities that result in marginalization, discrimination and stigma towards minority communities	• Trauma-informed approach • Culture change within hospital	• Education interventions • Improved culture with regards to WPV
Intersectional Identities [49]	• Intersectional identities outlines the power and control individuals experience in society due to different intersecting aspects of their identity including sex, gender, age, sexual orientation, ethnicity, race, religion, language, culture, education, presence of disability, geography, income, marital status, immigration status and indigenous status • Whether an individual experiences power or discrimination in their society is determined through the way their different identities are interpreted by the society they live in and the way their identities intersect, for example a person that identifies with multiple marginalize communities is more likely to experience discrimination than a person that identifies mainly with the identities in power • Individuals with marginalized identities possess more lived experiences of trauma are likely to feel less safe in social situations	• Societal risk factors	• Societal inequalities that result in marginalization of identities	• Trauma-informed approach • Culture changes within hospital	• Education interventions • Improved culture with regards to WPV

### Rationale

WPV in healthcare is an urgent issue that significantly impacts patients, chosen family members, healthcare providers (HCPs), and healthcare institutions (Fig. 1). The rising frequency and severity of WPV incidents, particularly in acute care settings, necessitate targeted interventions that address this pressing challenge [3, 4]. Research indicates that WPV not only harms individual well-being but also adversely affects overall quality of care, HCP morale and burnout, and contributes to an environment that compromises safety [53].

Previous attempts to address WPV have often fallen short due to the multifaceted nature of the problem [5, 7]. Research demonstrates that a multicomponent intervention utilizing a systems-based approach is necessary to effectively address WPV [5]. Consequently, a systematic framework outlining the identification and development of a complex WPV prevention intervention is essential in healthcare to assist organizations in creating comprehensive solutions to this complex issue.

Developing such interventions requires a robust QI initiative. QI projects in patient and staff safety often focus on the three categories derived from Donabedian’s Structure-Process-Outcome (SPO) model [54]; however, this approach omits a focus on the ‘human factor’ within the structure component and the individuals involved in the processes including patients, caregivers, chosen family members, HCPs and patient facing staff. To expand on the work developed by Donabedian, Carayon and colleagues released multiple iterations of the Systems Engineering Initiative for Patient Safety (SEIPS) [54–56]. With SEIPS, the “structure” component of Donabedian's framework is reimagined as a “work system”, where there are five main elements interacting: person, environment, organization, technology and tool, and task factors [54].

In this QI project, we incorporated the 3.0 iteration of the SEIPS framework to support us in developing a comprehensive, systems approach to address our multifaceted WPV challenges [56]. Further details on the operationalization of SEIPS 3.0 in our methods and interventions are provided in the Methods section.

### Specific aims

WPV in healthcare is a complex systemic issue that requires a comprehensive solution. The aim of this article is to present the development of a large-scale multifaceted QI project tailored to a multi-site academic health science centre in Toronto, Ontario. Additionally, we present a stepwise framework that summarizes our initial approach and learning points to support other healthcare institutions in developing an approach to address WPV from a systems theory lens.

## Methods

### Context

The Security Operations Program at a multi-site academic health science centre established a QI team to address WPV in November 2022. Our organization is a complex healthcare network comprised of three acute-care hospitals, including a cancer centre, a multi-site rehabilitation network and an educational institution. In 2022, this organization had a care volume of approximately 98,000 inpatient weighted cases, 1.1 million ambulatory visits, and 158,000 ED patients. WPV incidents in the ED increased overall during the COVID-19 pandemic (1.15 reported incidents per 1000 ED visits) compared to 2019 (0.43 reported incidents per 1000 ED visits), an increase of 169% (*p* < 0.0001). This data is consistent with data from other EDs globally [3, 4, 57]. The QI team consisted of healthcare professionals, administrative leaders, and researchers who were sponsored by the organization’s executive leadership, to develop a comprehensive strategy for ameliorating WPV. The QI initiative received formal Research Ethics Board exemption and approval from the organization's Quality Improvement Review Board (QI ID: 22–0499). Our reporting followed the Revised Standards for Quality Improvement Reporting Excellence guideline [58].

### Interventions

The SEIPS 3.0 framework guided a systemic approach to investigating and understanding the configurations of work system elements and functional entities, interactions through processes, and patient, caregiver, HCP, and organizational outcomes. SEIPS 3.0 was also selected as compared to past iterations of SEIPS as this iteration recognizes that patients move through various and unique work systems in their patient journey. Thus, SEIPS 3.0 provided us with a structure to examine factors influencing and interacting with WPV across multiple care environments.

Recognizing the need for practical tools to implement SEIPS, the creators developed a set of tools referred to as “SEIPS 101” [59]. These tools provide a simplified version of the SEIPS model that includes seven unique tools for QI initiatives [59]. We employed the SEIPS 101 tools in a variety of ways to identify areas for improvement regarding WPV at our institution. These identified areas informed the development of the subprojects that constitute our WPV QI project.

The first tool, the People, Environment, Tools, Tasks (PETT) scan is a checklist used to investigate the work system and provide the researcher with the broader context of the interactions within a system, it’s facilitators, barriers, contributing factors and areas of improvement [59]. A PETT scan was performed by collaborating with key stakeholders to complete the PETT scan template. The next tool is the people map, which takes the people aspect of a PETT scan to a more in-depth level. A people map identifies people involved in a work system, their roles, interactions with others, as well as the proximity of interactions [59]. To perform a people map, researchers must identify and document the roles, responsibilities, and relationships of individuals within a specific area or process. This involved gathering information from stakeholders then visually mapping out the connections and interactions [59].

Additional SEIPS 101 tools involve the use of matrices including Tasks and Tools matrices. This matrix identifies the tools that are being used for tasks within a work system and allows researchers to identify opportunities to streamline process and design or redesign tools to better serve the task [59]. We performed a tools and tasks matrix by engaging with key stakeholders and completing the SEIPS 101 Task Matrix, Tools matrix, and the Tasks X Tools Matrix ([59], Supplementary file, page 3]). We analyzed the matrix to identify gaps, redundancies, and areas for improvement, subsequently developing QI initiatives to optimize efficiency and effectiveness. Another tool includes the outcomes matrix, for each task or QI initiative, an outcome matrix can be performed to identify project goals, determining measures to be collected or establish evaluation criteria. We performed this task by completing an outcome matrix ([59], Supplementary file, page 4]), identifying desirable and undesirable outcomes as well as means to evaluate the outcomes.

The SEIPS 101 Journey map investigates the journey of an individual or functional unit throughout a process and identifies the impact and interactions with the work systems and its outcomes. The tool is valuable in highlighting issues or considerations when modifying processes or systems. We performed a journey map by documenting the step-by-step experiences and interactions of users throughout a process or system of interest. This enabled us to visualize the journey of the user throughout a process. The interactions diagram highlights the interactions between people, environments, tools, and tasks to identify areas for improvement and enhance understanding of the work system. To perform an interactions diagram, we mapped out the interactions between people, environments, tools, and tasks to visualize relationships and identify areas for improvement within the work system. Lastly, the systems story tool serves as a frame of reference by illustrating how changes in the work system impact processes and subsequent outcomes. This tool effectively communicates complex concepts and can be used to advocate for change. Researchers developed a narrative using findings from a qualitative interview study and employed storytelling techniques to engage stakeholders effectively. Further details of these tools can be found in the SEIPS 101 Supplementary file [59].

### Study of the Interventions

This article presents the development process of the 12 subprojects to provide a focused and detailed account of our QI interventions. For the purposes of this article, we will focus on presenting the development of the 12 subprojects, outlining how SEIPS 3.0 and SEIPS 101 tools informed the creation of these interventions. This approach provides a detailed account of the development process of the interventions. Future publications will provide a presentation of the effectiveness and outcomes of these interventions.

## Results

### Identifying where change is needed within UHN

**Fig. 2:**
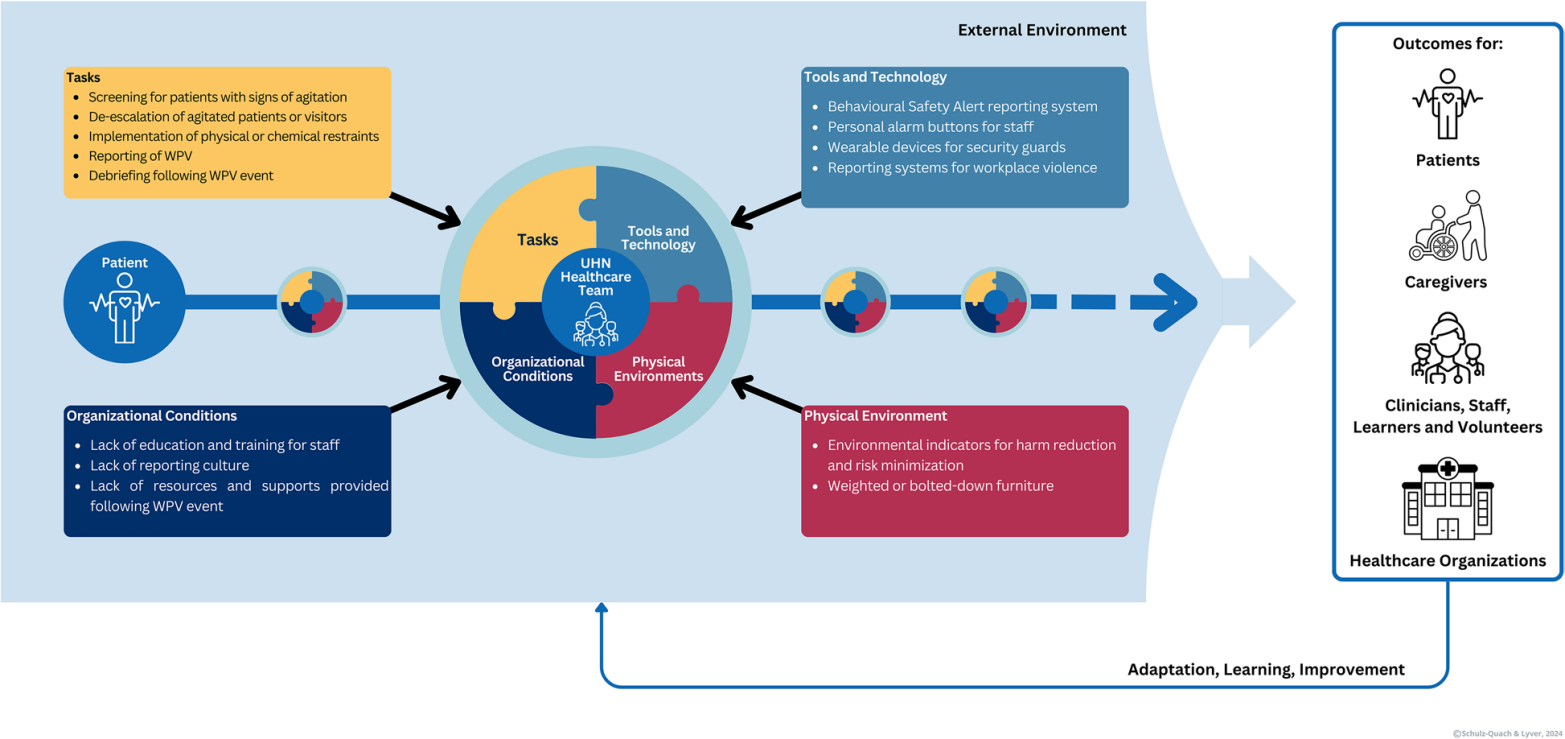
An illustration of the application of the SEIPS 3.0 journey map to identify the tasks, tools and technology, physical environment, and organizational conditions relevant to WPV in healthcare. Adapted from [56]

The SEIPS 3.0 framework and SEIPS 101 tools guided our environmental scan and strategic planning with stakeholders in order to develop an initiative aiming to collectively understand, measure, and ameliorate WPV at our organization. A SEIPS 3.0 journey map was created by our QI team to outline the key factors and interactions related to WPV within the work system. The QI team collaborated with 48 stakeholders that were identified as experts who are involved in some aspect of WPV [60] to gather information on workflows and WPV related processes in 5 meetings, in addition the QI team reviewed existing data related to WPV. This information was used to map the patient journey as it relates to WPV, identifying the various people, tasks, tool and technologies, as well as organizational and environmental conditions related to WPV (Fig. 2). This map provided the foundation for our QI project, guiding the team in identifying necessary areas to investigate using the SEIPS 101 toolbox.

After identifying key intervention areas through the SEIPS 3.0 journey map, the QI team selected specific SEIPS 101 tools to further investigate these areas and guide the development of our QI interventions. Ultimately, a comprehensive QI initiative consisting of 12 subprojects was developed and implemented based on insights gained from the application of the SEIPS 101 tools (Table 3). To illustrate our approach, this article will focus on three example topic areas drawn from these subprojects.

**Table 3:** Summary of the 12-subproject quality improvement initiative, including the application of SEIPS 101 tools and proposed outcome measures for each subproject. For additional information on the 12 subprojects, please refer to Appendix 1

**Subproject number**	**Subproject**	**Subproject description**	**SEIPS 101 tools utilized**	**Structural units responsible**	**Associated change implemented**	**Proposed outcome measure**
1	Ongoing Literature Review of WPV-related topics	Ongoing literature reviews of current research pertaining to WPV in healthcare were performed to provide UHN Security with the most up to date information necessary to make informed decisions.	• Outcomes Matrix	• UHN Security WPV QI Team	Ongoing literature reviews on the following topics related to WPV: • WPV in healthcare settings during the pandemic • Agitation Management • De-escalation Techniques • Code White Simulation Training	• number of hot debriefs since implementation • number of cold debriefs logged since implementation • number of Code White/WPV requests for escalation
2	Identifying quality indicators for measuring change in quality of care related to Code White and WPV incidences	Based on a rapid review, a collated list of quality indicators will be condensed to the top relevant, impactful and feasible indicators through an anonymous modified Delphi process. This process will utilize experts from UHN and Toronto Academic Health Science Network.	• Outcomes Matrix	• UHN Security WPV QI Team	• Rapid review of quality indicators to measure WPV-related processes in healthcare • Modified Delphi process to select top indicators	• number of governance committee meetings • number of annual progress reports
3	Implementing quality indicators to an organization-wide WPV dashboard	A WPV dashboard with the top evidence-based quality indicators identified from the modified Delphi process will be created to measure WPV at UHN and be used to inform leadership decision making.	• Outcomes Matrix • Journey Map	• UHN Security WPV QI Team	• WPV dashboard creation	• number of hot debriefs since implementation • number of cold debriefs logged since implementation • number of Code White/WPV requests for escalation
4	Changing the perception of safety and support in HCPs during Code White and WPV incidences	A longitudinal qualitative approach to capture ED staff’s perception of safety, support, clinical guidance relating to managing and learning from WPV incidence, security and personal expectations for organizational change. Additionally, measuring change over time in sense of preparedness to handle WPV situations using Bandura’s self-efficacy theory.	• Systems Story	• UHN Security WPV QI Team	• Performing semi-structured qualitative interviews with all staff, learners and volunteers in the ED on an ongoing basis	• number of meetings regarding community outreach
5	Implementation of educational intervention I	An ad-hoc training program focused on situational and environmental awareness was implemented as a pilot training program for ED staff. The program would provide staff with additional training methods and skills for safety and self-protection while determining whether a need for additional training was evident.	• Outcomes Matrix • Systems Story	• UHN WPV Education Collaboration • UHN Safety Services • Code White Governance Committee • UHN Security WPV QI Team	• Implementation of a pilot WPV skills training session	• number of meetings regarding reporting systems
6	Implementation of a dedicated UHN Code White Governance Committee	A governance committee dedicated to Code Whites at UHN that will be responsible for the following tasks: • establishing a streamlined approach and response to Code White incidents • overseeing implementation of high complexity Code White simulation training that uses a trauma-informed lens • optimize Code White incident reporting, including a user-centred and trauma-informed reporting approach • optimizing of auditing statistics and quality indicators for Code White clinical care at UHN	• PETT Scan • People Map • Journey map • Interactions Diagram	• UHN WPV Prevention Advisory Committee • Code White Governance Committee	• Implementation of an interdisciplinary Code White Governance Committee	• number of hot debriefs since implementation • number of cold debriefs logged since implementation • number of Code White/WPV requests for escalation
7	Reviewing, updating and implementing incident reporting for WPV and Code White incidents	A working group made up of multiple functional units at UHN are reviewing the incident reporting processes and reporting systems at UHN. The working group will update UHN’s reporting system to develop a streamlined, user-centred and trauma-informed reporting approach.	• Tools and Tasks Matrix	• UHN Quality and Safety • UHN Safety Services • UHN Emergency Preparedness • Code White Governance Committee	• Development of Safety Event Reporting and Review System (SERRS) Project	• number of meetings on physical restraint systems
8	Implementation of educational intervention II (UHN TIDES)	The development of a trauma-informed WPV prevention education program centred around agitation management, de-escaltion techniques, physical safety, self-protection and code white simulation. The training must be specific to staff’s environment, provide the opportunity for different positions to work together and provide refresher training.	• Outcomes Matrix • Systems Story	• UHN WPV Education Collaboration • UHN Safety Services • Code White Governance Committee • UHN Security WPV QI Team • External Collaboration partner	• Implementation of UHN TIDES	• number of articles related to WPV in healthcare that were reviewed
9	Implementing Environmental Indicators for harm reduction and risk minimization	Environmental signage using trauma-informed language to communicate a message of mutual respect between hospital staff and visitors to create an environmental and cultural change surrounding WPV in EDs.	• Interactions Diagram	• UHN Security WPV QI Team	• Implementation and evaluation of environmental indicators for mutual respect in UHN EDs	• number of quality indicators identified from the literature • number of unique quality indicators operationalized for UHN • number of unique quality indicators selected through the Delphi Process
10	Code White hot and cold debriefing process and debriefing escalation algorithm	A new approach to debriefing following Code White and WPV events will be implemented using hot and cold debriefs. Additionally, an algorithm for escalating a Code White or WPV event will be developed.	• PETT Scan • People Map • Tools and Tasks Matrix • Journey Map • Interactions Diagram	• UHN Emergency Preparedness • Code White Governance Committee • UHN Security WPV QI Team	• Implementation of hot and cold debrief guidelines • Implementation of new communication strategy for entities requesting cold debriefs	• number of quality indicators implemented • number of databases used to provide data
11	Physical restraint systems	Currently no guidelines or policies on physical restraints are provided on a provincial or national level. A descriptive physical restraint system is required for a universal approach to applying physical restraints.	• Tools and Tasks Matrix	• Code White Governance Committee • UHN Security WPV QI Team	• Development of physical restraint system	• number of staff that completed training program • evidence of staff satisfaction with training
12	Patient partners and community outreach	Connecting with patients, caregivers and (chosen) family members and providing them with the opportunity to connect and talk about their experience during Code White or WPV events.	• People Map • Systems Story	• Code White Governance Committee • UHN Security WPV QI Team	• Implementing a town hall style meeting with patients, caregivers and (chosen) family members involved in Code White or WPV events	• evidence of new training program • number of trainers hired

### Code white interventions: subprojects 6 and 10

Multiple SEIPS 101 tools were employed to investigate Code White incidents and related processes at UHN, including a PETT scan, people map, tools and task matrix, journey map, and interactions diagram. These tools facilitated a comprehensive understanding of the Code White process and informed the development of targeted interventions.

In collaboration with 10 key stakeholders, including representatives from Safety Services, the Emergency Department, and the Centre for Mental Health, we conducted a PETT scan using the PETT scan template to identify the people, environments, tools, and tasks involved in a Code White incident. Following this, a people map was created to further examine the roles of individuals and functional units engaged during a Code White (Table 4).

**Table 4:** Functional units at UHN addressing or involved in processes related to WPV and Code White incidents within UHN [2]

**Code White Governance Committee (CWGC)**	• Composed of clinical and academic experts in the field of agitation management
	• Provide guidance and structure required to streamline and optimize CW management
**Workplace Violence Prevention Advisory Committee (WPVPAC)**	• Put in place by the UHN executive leadership forum to address WPV across UHN
**Workplace Violence Education Collaboration (WPVEC)**	• Collaboration between Safety Services and Security that is providing staff, learners and volunteers with education related to WPV and CWs
**Quality of Care Committee**	• Involved in reviewing and improving the quality of care provided at the healthcare institution
**Safety Services**	• Prioritizes and monitors the safety of UHN staff and patients
**Emergency Preparedness (EP)**	• Supports UHN in preventing, mitigating, preparing for and recovering from emergency events that impact staff, patient and visitor safety and the delivery of critical services
**Facilities Management-Planning, Redevelopment and Operations (FM-PRO)**	• Responsible for a wide range of tasks related to maintaining the physical environment at the healthcare institution
**Security Operations**	• Responsible for providing UHN staff and patients with protection, security and support

**Fig. 3:**
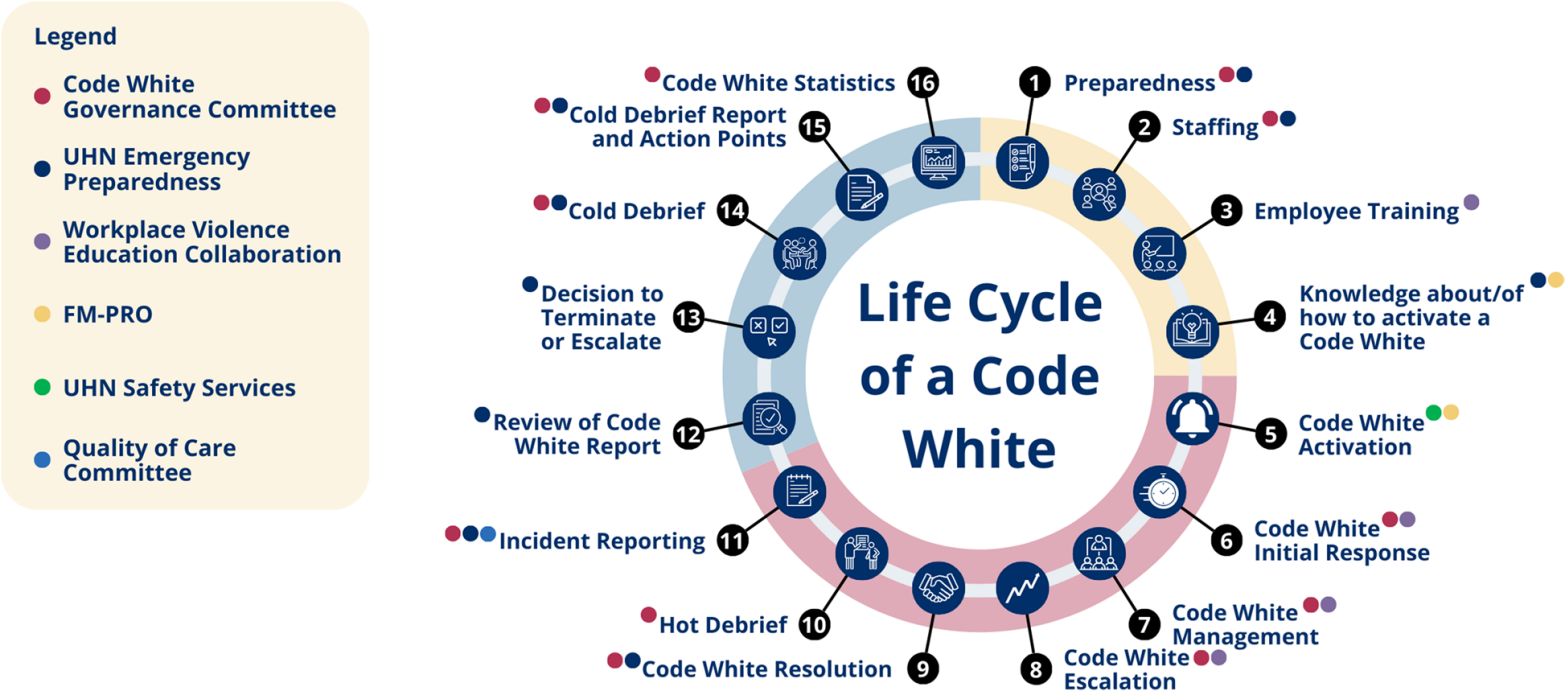
The Life Cycle of a Code White, a journey map that outlines the steps involved in a Code White and the functional units involved [2]

We then combined an interactions diagram with a journey map to develop the Life Cycle of a Code White (LCCW) (2). The information gathered from the PETT scan and people map was used to outline all steps in a Code White and identify the functional units involved at each stage (2). The LCCW revealed 16 distinct steps and the participation of 6 functional units, highlighting the complexity of Code White incidents (Fig. 3). This comprehensive understanding underscored the need for a governance committee to evaluate and streamline Code White processes. As a result, these SEIPS 101 tools collectively identified the need to implement a Code White Governance Committee (CWGC) (subproject 6).

Furthermore, the LCCW identified the need to streamline our organization’s debriefing approach. Our QI team collaborated with Emergency Preparedness to perform a tools and task matrix for the debriefing process. Following this task, a new Code White hot and cold debriefing process and debriefing escalation algorithm would be developed (subproject 10).

### Education interventions: subprojects 5 and 8

**Fig. 4:**
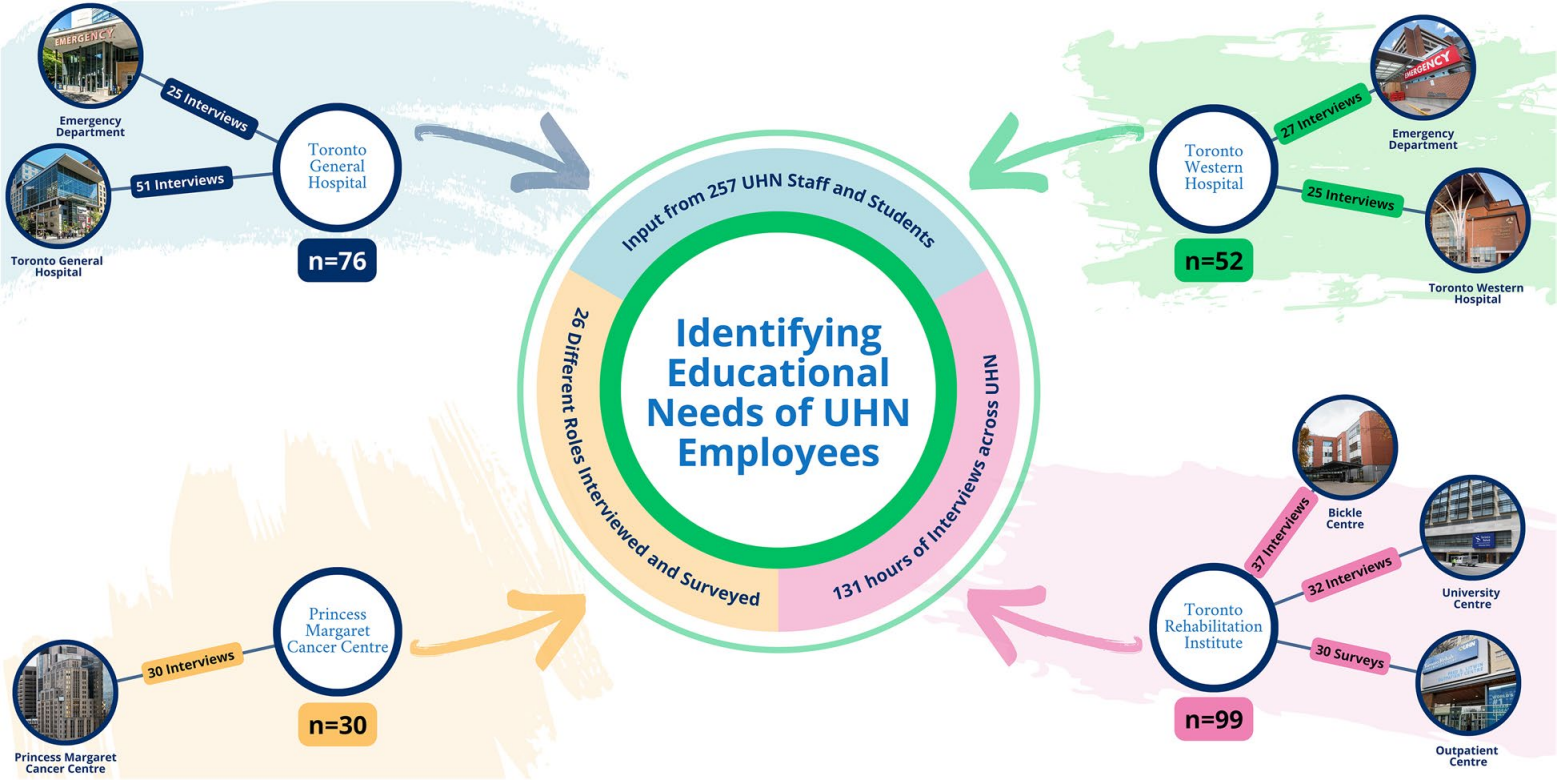
Overview of the educational needs assessment conducted at UHN. The assessment collected input from 257 UHN staff and students from 26 different roles across all sites. The data collected was used to create a systems story

A need for education interventions was identified and developed using an outcome matrix and the systems story tool. Both tools were informed by qualitative data (Appendices 2 and 3), which was collected through semi-structured interviews conducted by our QI team at both UHN EDs (*n* = 52) and by the CWGC, which conducted 175 interviews and 30 surveys across all UHN sites (Fig. 4). All interviews and surveys underwent a thematic analysis performed by our QI team and the CWGC. The findings of the analysis frequently were related to staff feeling unsafe, unsupported and emphasized a need for education related to WPV prevention. A detailed summary of the findings will be presented in a separate publication.

An outcome matrix was developed by our QI team based upon the findings of the thematic analysis. Project goals included improving staff’s sense of safety, support and confidence in de-escalation. The matrix allowed us to identify that education interventions across the organization were needed, however, implementing such a project in a large organization would require executive level support. Consequently, the thematic analysis was utilized to illustrate a systems story that effectively advocated the need for WPV prevention education to UHN leadership. The systems story helped advocating with leadership to implement an ad-hoc education initiative (subproject 5) was implemented as a pilot project, in addition to supporting implementation and rollout of an organization-wide WPV prevention education program (subproject 8).

### Environmental interventions: subproject 9

**Fig. 5:**
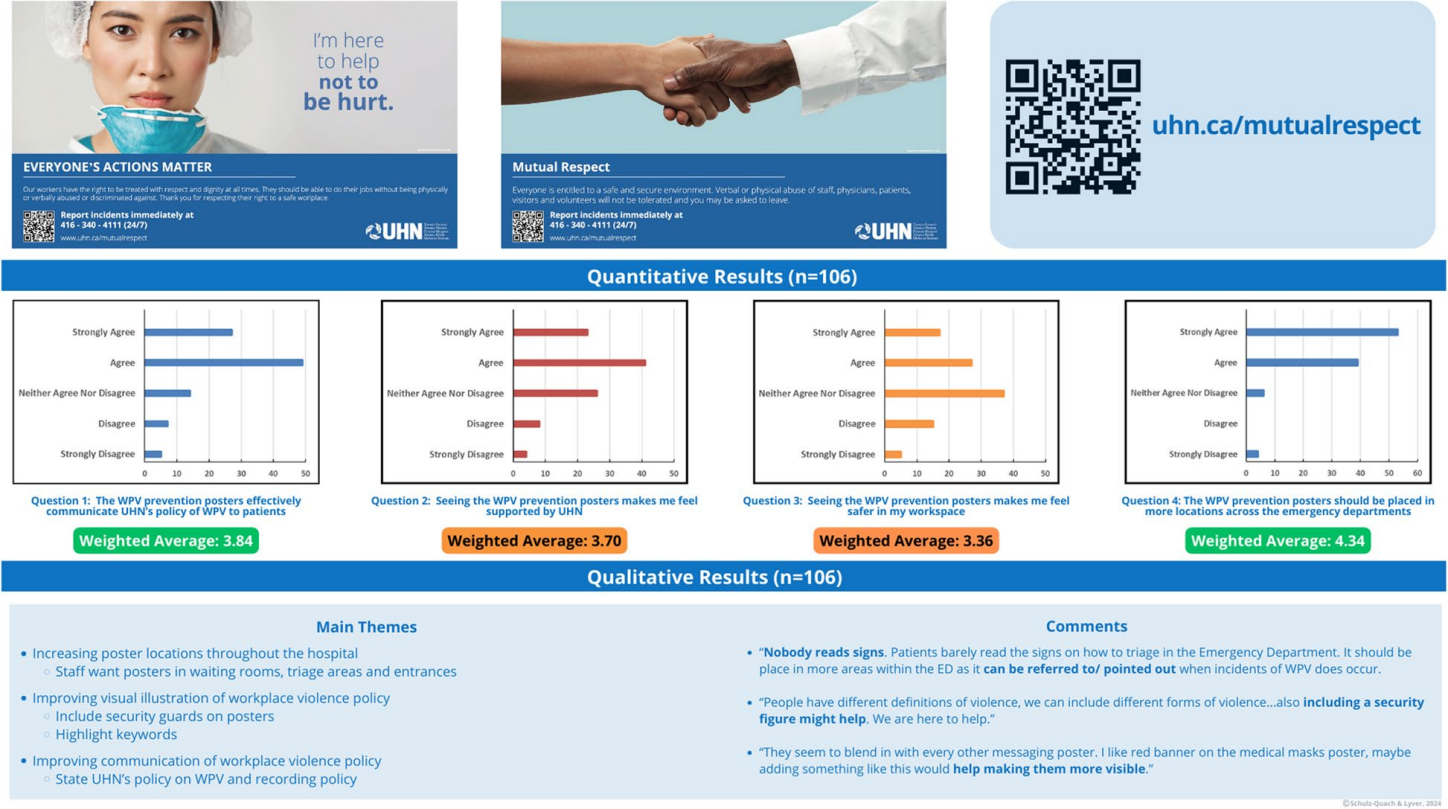
An overview of the Environmental Indicators Project, featuring samples of the posters utilized, alongside qualitative and quantitative feedback obtained from staff through surveys

An interactions diagram was utilized to explore the relationships between people, environments, tools, and tasks during a WPV incident, with the aim of identifying areas for improvement within the work system. Utilizing the lived experiences of our QI team members as clinicians, code white respondents, and security personnel, we mapped these interactions to better understand the dynamics involved. The diagram revealed the absence of an environmental component that actively advocated for WPV prevention. Utilizing findings from an environmental scan of other healthcare and public service institutions, such as public transit, we identified posters as an effective tool for communicating messages of mutual respect and hospital policies related to WPV. In response, five posters were developed using trauma-informed language to promote mutual respect between patients and HCPs (Fig. 5). Feedback was gathered from 106 staff members through surveys (Appendix 4) using a convenience sampling method. Although staff voiced that the posters were not enough to prevent WPV, they supported the project and requested that more posters be placed throughout the hospital (Fig. 5). Further details from these surveys will be shared in a future publication.

## Discussion

### Summary

In summary, a large-scale QI initiative was developed using SEIPS methodology to arrive at 12 subprojects at our large multi-site academic health sciences centre in November 2022 with the objective of addressing WPV from a systemic perspective. The project consisted of various literature-based, community, educational, and organizational interventions that were identified and informed using the SEIPS 3.0 framework and SEIPS 101 tools.

### Interpretation

#### Systematic framework to address WPV in healthcare institutions

The following framework was created to provide guidance on how to approach WPV in a healthcare institution. Although, there is an abundant amount of literature on WPV-related topics and interventions, in addition to frameworks for QI projects, there is limited guidance on how to address WPV in hospital settings. Therefore, we created a 12-step framework which was also used to guide our WPV QI initiative at UHN (Table 5).

**Table 5:** Summary of the 12 Steps in our systematic framework for addressing WPV in healthcare institutions, including the associated subprojects implemented at our healthcare institution for each step

**Framework step number**	**Framework step**	**Step description**	**Evidence**	**Corresponding subproject**
1	Define the Problem and Find the Data	WPV quality improvement projects should begin by clearly defining the problem and reviewing available data. Tools like the SEIPS 101 PETT scan help identify key elements and interactions within the work system, including barriers, risk factors, and facilitators, providing a comprehensive understanding of the WPV problem. This method is particularly useful for organizations that lack sufficient data due to issues like underreporting.	• Holden & Carayon, 2021 • Chung et al., 2014 • Cumbler et al., 2013 • Bokhoven et al., 2003 • Mento et al., 2020	SP1. Ongoing Literature Review of WPV-related topics SP3. Implementing quality indicators to an organization-wide WPV dashboard SP4. Changing the perception of safety and support in HCPs during Code White and WPV incidences
2	Assemble a WPV QI team	Addressing WPV is a complex task that will require a well-rounded interdisciplinary team. The team should include individuals with diverse perspectives, skill sets, and backgrounds and should emphasize collaboration and creativity in order to have effective WPV quality improvement.	• Chung et al., 2014 • Kaplan et al., 2012 • Hulscher et al., 2013	This relates to the approach taken for the systems level QI project.
3	Listen to Frontline Staff	Collecting qualitative data from frontline staff is crucial for WPV initiatives. WPV contributes to HCP burnout, high turnover rates, and reduced quality of care; ensuring HCPs feel heard fosters a sense of organizational support and yields valuable insights. Integrating both qualitative and quantitative data through interviews and surveys enables organizations to better understand WPV, support staff, and guide QI efforts. A mixed-methods approach, including longitudinal data, enhances intervention effectiveness and secures key collaborators' buy-in.	• Schulz-Quach et al., 2022 • Shanafelt et al., 2020 • Crowe et al., 2017 • Calman et al., 2013 • Sachdeva et al., 2007 • Franco et al., 2011	SP4. Changing the perception of safety and support in HCPs during Code White and WPV incidences
4	Key Collaborator Engagement	Active engagement with key collaborators, especially organizational leadership, is essential for the success of WPV QI projects. Leadership buy-in ensures resource availability, fosters cultural change, and supports the sustainability of ongoing WPV initiatives like training. Early involvement, clear communication, and including key collaborators in decision-making are critical to maintaining their long-term support.	• Kaplan et al., 2012 • Fryer et al., 2007 • Kaplan et al., 2010 • Brandrud et al., 2011 • Guise et al., 2013	This relates to the approach taken for the systems level QI project.
5	Bringing Organizational Entities Together	Addressing WPV requires collaboration beyond the QI team, involving representatives from all functional units to ensure a comprehensive understanding of the problem. However, siloed operations in healthcare institutions often hinder efficiency and can undermine organization-wide initiatives like WPV prevention. Effective communication between departments must be established early, as demonstrated in our case, where eight distinct functional units addressing WPV were identified and brought together before initiating change.	• Chung et al., 2014 • Alves et al., 2018 • Akmal et al., 2021	This relates to the approach taken for the systems level QI project.
6	Implement an Effective Governance Structure	An effective WPV governance structure is vital to project success, providing leadership, preventing conflicts, managing resources, and ensuring sustainability. In our large organization, multiple governance structures were developed alongside existing departments to support the WPV QI initiative. The governance framework should include leadership from all relevant functional units, with a clear charter outlining roles, budgeting, goal alignment, and data sharing. Additionally, presenting to senior executives is essential for securing full organizational endorsement.	• Kaplan et al., 2012 • Fryer et al., 2007 • Derakhshan et al., 2019 • Jones et al., 2017	SP6. Implementation of a dedicated UHN Code White Governance Committee
7	Assess Project viability and Monitor Progress and Engagement of Functional Units	Assessing viability and monitoring project execution are crucial for multi-level WPV initiatives to achieve successful outcomes. The DICE framework, developed by the Boston Consulting Group, evaluates key factors such as project duration, team integrity, commitment of senior executives and frontline collaborators, and the additional effort required from staff. Continuous monitoring helps maintain engagement and commitment, particularly in the face of delays and resource constraints, ensuring the sustained progress of WPV initiatives.	• Hughes, 2021 • Sirkin et al., 2005 • Day et al., 2017 • Raza et al., 2023 • Ziółkowski et al., 2006	This relates to the approach taken for the systems level QI project.
8	Connect with the Community	In addressing WPV, it was crucial to include patients, chosen family members, and visitors, as they experience various stressors during healthcare visits that can trigger stress responses, minority stress, and responsive behaviour, potentially leading to WPV incidents. A trauma-informed and inclusive approach is necessary to create a safe environment for these groups. Engaging with these groups through surveys, advisory boards, and involvement in developing initiatives offers valuable perspectives on WPV. It is essential to foster meaningful and authentic engagement to avoid feelings of under appreciation or tokenism among patients.	• Muskett, 2014 • Beattie et al., 2019 • Schulz-Quach et al., 2023 • Ashworth et al., 2023 • Armstrong et al., 2013 • Baker et al., 2016 • McNeill et al., 2020	SP12. Patient partners and community outreach
9	Implement a Cohesive and Clear Communication Strategy	Effective organizational communication is crucial for the success of WPV QI initiatives. Clear and cohesive communication from leadership helps staff understand the organization's direction, which enhances HCP buy-in and engagement. Inconsistent communication can lead to rumours and create divisions that undermine cohesiveness and trust. Successful WPV initiatives can be shared through various channels, including newsletters, emails, websites, meetings, and leadership updates. Utilizing existing communication strategies increases effectiveness, and establishing a communication channel between site managers and the WPV QI team allows for valuable site-specific feedback.	• Boan & Funderburk, 2003 • Brown, 2020 • Seijts & Crim, 2006 • Simmonds, 2006 • Agency for Healthcare Research and Quality, 2017 • Kellogg et al., 2017	SP9. Implementing Environmental Indicators for harm reduction and risk minimization
10	Implement Data Monitoring and Utilize Statistics for Planning/Management Decisions	Measuring changes in regions of interest related to WPV over time is essential for assessing the impact of WPV QI initiatives. However, current WPV metrics in healthcare often focus solely on outcome indicators, such as the frequency of documented incidents, which are problematic due to the historical underreporting of WPV. To effectively monitor WPV, healthcare institutions need a broader set of quality indicators that encompass structure, process, and outcome measures, providing a comprehensive view of WPV within the organization.	• Keith & Brophy, 2021 • Lyver et al., 2024 • Sethi et al., 2024 • Byon et al., 2022 • Itri et al., 2017	SP2. Identifying quality indicators for measuring change in quality of care related to Code White and WPV incidences SP3. Implementing quality indicators to an organization-wide WPV dashboard
11	Improve Debriefing and Reporting	Improving debriefing and reporting protocols in healthcare institutions enhances HCP well-being and organizational culture, which are vital for increasing buy-in and incident reporting. Effective debriefing minimizes adverse outcomes by providing support and validation without placing blame. Addressing the underreporting of WPV, a consistent issue in healthcare, requires a robust, accessible reporting system that minimizes staff workload and includes follow-up communication. Education and debriefing interventions should promote these reporting systems to foster a culture of reporting, ensuring staff feel supported and cared for.	• Shanafelt et al., 2020 • Antai-Otong, 2001 • Fricke et al., 2023 • Juarez, 2021 • Rodrigues et al., 2021 • Arnetz, 2022 • Johnson, n.d. • Thomas et al., 2021	SP7. Reviewing, updating and implementing incident reporting for WPV and Code White incidents SP10. Code White hot and cold debriefing process and debriefing escalation algorithm
12	Implement Comprehensive Training Plan based on HCP’s Environmental Risks	Implementing a new or updated training plan tailored to staff needs is essential for addressing WPV in healthcare. Effective training enhances WPV management, increases staff safety, and fosters a culture of safety within the organization. Training should encompass simulation and education programs focused on WPV awareness, de-escalation, agitation management, decision-making, crisis intervention, and conflict resolution. Since factors such as department, patient interaction frequency, and WPV concerns influence the likelihood of involvement in incidents, training should be based on risk profiles rather than solely on profession. This approach fosters interdisciplinary understanding, enhances interprofessional communication, and improves teamwork.	• Keith & Brophy, 2021 • Beech & Leather, 2006 • Martinez, 2017 • Alafean & Dalahmeh, 2022 • Liu et al., 2020 • Walton et al., 2019	SP5. Implementation of educational intervention I SP8. Implementation of educational intervention II

### Step 1: define the problem and find the data

WPV quality improvement projects begin with properly defining the problem and finding reviewing the available data. Identifying the root causes of problems is critical to preventing issues from reoccurring. There are multiple problem-analysis methods to define problems such as the Ishikawa fishbone diagram or root cause analysis [61, 62]. Regardless of the method used, it is important to define the WPV problem, identify barriers, risk factors, contextual factors and facilitators of change [62, 63]. The PETT scan from the SEIPS 101 toolbox provided us with an effective method of investigating the people, environment, tools, tasks and the interactions between them in a work system to assist with defining our WPV problem [59]. The PETT scan is a checklist that encourages users to identify a comprehensive understanding of key elements interacting within the work system, as well as the barriers and facilitators of each work system component. Using these data excavation tools will assist healthcare organizations in identifying where WPV interventions are required. Utilizing SEIPS 101 tools at the commencement of a WPV QI initiative can further be helpful if healthcare institutions lack the data necessary to demonstrate an increase in WPV due to common barrier factors such as underreporting [64].

### Step 2: assemble a WPV QI team

An important preliminary step in WPV QI is assembling an effective team. An interdisciplinary team approach supports leveraging diverse perspectives, skillsets, and knowledge of team members with various backgrounds while fostering opportunities for collaboration and creativity necessary for effective WPV quality improvement [61]. When forming a QI team, it's important to consider contextual factors that have been shown to contribute to success including team diversity, physician involvement, subject matter experts, team members with a history of working together, prior experiences and skills with QI, leadership and a sound decision-making process [65, 66]. Assembling a well-rounded QI team mindful of these factors sets the foundation for a successful and collaborative QI initiative addressing WPV in healthcare.

### Step 3: listen to frontline Staff

While it is always essential to ensure voices of frontline staff are heard, this becomes particularly crucial when addressing WPV in healthcare. WPV is a demoralizing issue contributing to undesirable patient outcomes, HCP burnout and high turnover rates, thus, making HCPs feel heard on this subject by their own organization can have a positive impact on staff engagement with the WPV QI process [67]. Performing qualitative interviews and collecting data through pulse surveys with frontline staff throughout the project is crucial. HCPs need to be heard, supported, and cared for, and prepared for the envisioned change, and ultimately, be protected within their healthcare settings, as has been discussed in a recent study in the context of the collective impact of the COVID-19 pandemic [68]. WPV-related qualitative data, complementing quantitative data, proves pivotal in addressing intricate issues and guiding WPV QI initiatives [69]. Employing a longitudinal approach supports the identification of lived experience of change, impact or lack thereof overtime [70]. Lastly, meaningful integration of both qualitative and quantitative data enhances the probability of securing key collaborators buy-in [71, 72]. By employing a mixed-methods methodology, teams can combine both quantitative and qualitative data which is often needed to pragmatically address WPV challenges.

### Step 4: key collaborator engagement

A critical phase in QI projects involves active engagement with key collaborators. Securing organizational leadership buy-in is a prerequisite for any project success, as it ensures the availability of resources [73]. This includes funding and protected time for team members which is crucial for success in WPV QI projects [74]. Involving leadership is pivotal in creating a cultural change that fosters supportive behaviours of WPV QI initiatives within the organization [65]. Additionally, many WPV QI initiatives, such as training, are ongoing and require ongoing funding commitment. Maintaining engagement of key collaborators is crucial to ensure that these WPV QI initiatives are sustained [75]. Research demonstrates that engaging key collaborators early, maintaining ongoing clear communication with key collaborators and involving key collaborators in project decision making are effective means of maintaining key collaborators involvement and support [76].

### Step 5: bringing organizational entities together

With WPV being a systemic concern, collaboration must continue beyond the QI team. In most healthcare institution, several functional units are likely to be involved in processes related to WPV, collaborating with representatives from all functional units involved in these processes is necessary to gain comprehensive problem understanding [61]. Unfortunately, functional units in healthcare institutions often operate with silo effects that compromise efficiency and promote conflict, repeated initiatives and sometimes ineffective use of resources [77]. Silo mentality is particularly harmful to organization-wide QI initiatives such as addressing WPV [78], thus proper communication between hospital departments needs to be established from the start. In our case, when investigating functional units addressing WPV using a people map, eight separate functional units involved in WPV were identified (Table 4). It was imperative that these organizational entities were brought together first before initiating change processes.

### Step 6: implement an effective governance structure

**Fig. 6:**
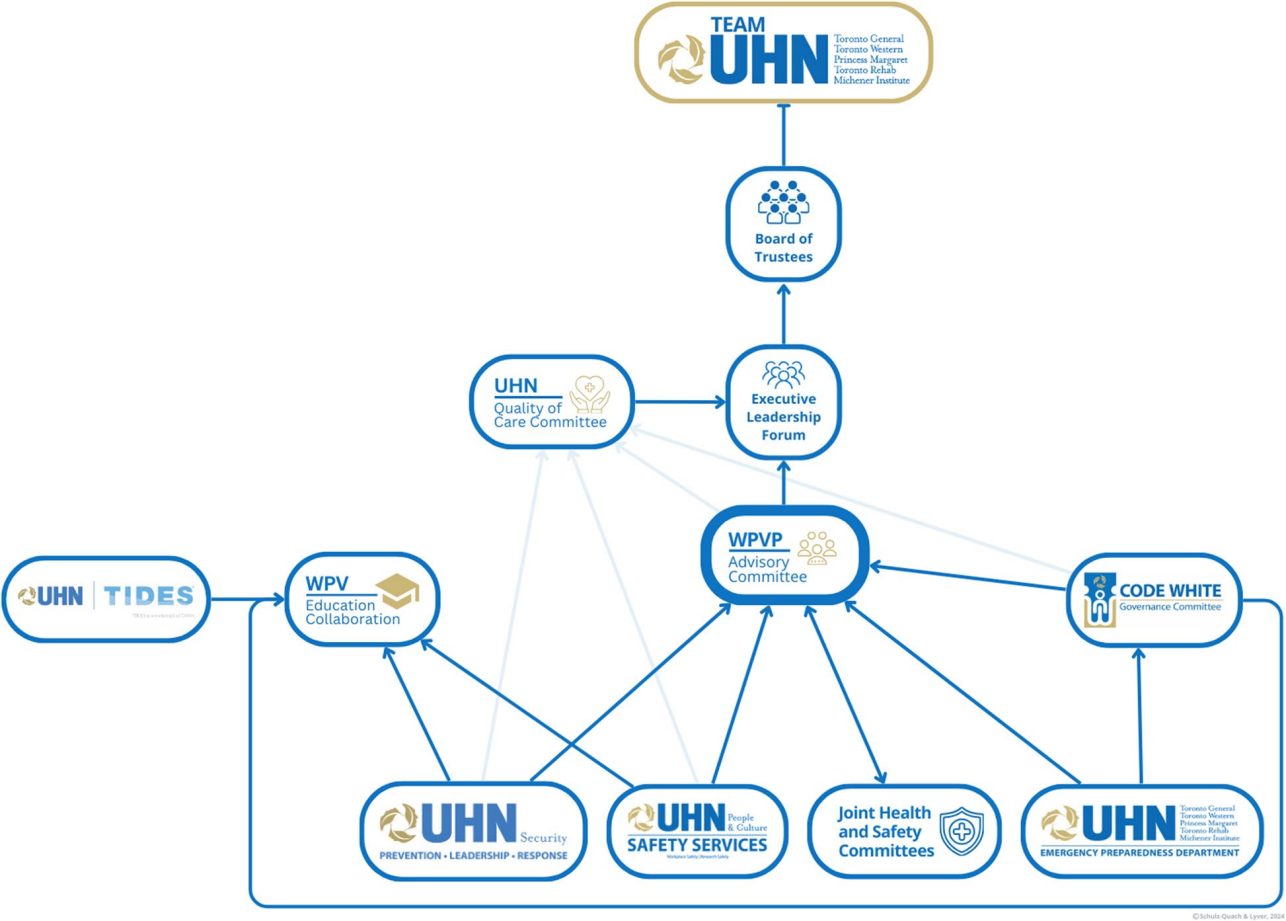
The WPV governance structure implemented at our organization to orchestrate a systemic approach to addressing WPV

Once all involved partners are identified, an effective WPV governance structure is crucial to project success [79]. This offers the leadership and management essential to prevent conflicts in project implementation, resource management, and ensure sustainability [65]. Given the size and complexity of our organization, multiple governance structures were developed in conjunction with existing departments and committees to ensure the success of a large-scale WPV QI initiative (Fig. 6). The governance structure should include leadership representation from all functional units involved in WPV initiatives or related processes. A charter for rules and roles of each member should include budgeting analysis, goal alignment and prioritization, data sharing for QI, as well as the timeline of projects [80]. Furthermore, it is imperative that the governance framework has the opportunity to present to the senior most executive level of the organization to ensure securing comprehensive organizational endorsement for its initiatives [73].

### Step 7: assess project viability and monitor progress and engagement of functional units

It is imperative to assess viability and monitor project execution by a governance team for multi-level projects to reach successful outcomes [81]. A validated tool can reduce resource waste [82], which is crucial for assessing the viability of a WPV initiative. Additionally, continuous project monitoring is necessary to maintain employee engagement and commitment, factors that can be influenced by burnout [83, 84]. The Boston Consulting Group has developed an efficacious four-element model known as Duration, Integrity, Commitment and Effort (DICE), [82, 85]. This framework can highlight important determinants of program viability such as the duration of the initiative or sub-projects, the integrity and skills of the team, the commitment level of senior executives and front-line key collaborators, and the additional effort required from the workforce. As subprojects will have natural delays and competing resources, strategic resource allocation and monitoring of functional units is crucial to the continuous progression of WPV initiatives.

### Step 8: connect with the community

In addition to placing a focus on HCPs and frontline staff, it was important to us to include patients, (chosen) family members and visitors when addressing WPV. Each group is subjected to numerous stressors during visits to a healthcare institution, a multitude of factors that impact their experience at the healthcare institution can trigger stress responses, minority stress and responsive behavior which can increase the probability of WPV events [86]. Trauma-informed care and inclusive lens is a requirement to ensure that patients, (chosen) family members and visitors can feel as safe as possible [9, 87, 88]. Engaging with patients involved in past WPV or Code White incidents provides a different and often complementing perspective of WPV events. However, there exists challenges in reaching out to patients and visitors such as patients feeling underappreciated, unheard and that the gesture is tokenistic [89]. Research has demonstrated that including patient advisors in the development of initiatives, collecting information from patients and visitors on their experiences via surveys and developing patient and visitor advisory boards are effective methods of engaging with patients, (chosen) family members and visitors [90, 91].

### Step 9: implement a cohesive and clear communication strategy

Ensuring organizational communication regarding WPV QI initiatives is imperative to project success [92]. Clear and cohesive communication from organizational leadership is essential for ensuring staff members understand the organization's direction, leading to increased HCP buy-in and engagement [93, 94]. A lack of consistency in intra-organizational communication can result in rumours and a divide between individuals or groups with knowledge and without that negatively impacts cohesiveness and organizational trust [95]. WPV QI initiatives and successes can be communicated through organization-wide newsletters, emails, websites, office computer screens, meetings, in-person handouts and leadership communication [96]. Researchers demonstrate utilizing pre-existing organization communication strategies improves effectiveness [97]. Developing a communication stream between site managers and WPV QI team members is another valuable tool that provides the opportunity for managers to provide site-specific feedback on WPV initiatives [97].

### Step 10: implement data monitoring and utilize statistics for planning/management decisions

Measuring changes in regions of interest pertaining to WPV overtime is pivotal to monitoring the impact of WPV QI initiatives. However, WPV metrics at healthcare institutions often place an emphasis exclusively on outcome indicators including the frequency of documented WPV incidents [98]. These indicators are problematic as WPV is historically underreported in healthcare [7]. Consequently, healthcare institutions will require a larger set of WPV quality indicators that do not rely solely on staff reporting of incidents to successfully monitor WPV [60]. These quality indicators must include structure, process and outcome measures to capture a comprehensive and systemic perspective on WPV within an organization [8, 99]. In our case example, we performed a rapid review and Delphi process to determine quality indicators that would provide the quantitative data imperative to monitoring WPV QI impact and for informing decision making [60].

### Step 11: improve debriefing and reporting

Enhancing debriefing and reporting protocols in healthcare institutions has been demonstrated to improve HCP well-being, and organizational culture [100, 101] both of which are pivotal to increasing HCP buy in and reporting of WPV incidents. Debriefing after WPV incidents minimizes adverse outcomes to staff and provide them with a sense of support, connectedness, and relief following the event [100, 102]. However, debriefing must not leave HCPs feeling blamed or criticized, a positive debrief checks in with staff, validates their feelings and encourages help-seeking when needed [103]. Utilizing a protocol for debriefing after WPV events enhances quality and consistency of debriefs in order to meet the support needs of HCPs [100].

**Fig. 7:**
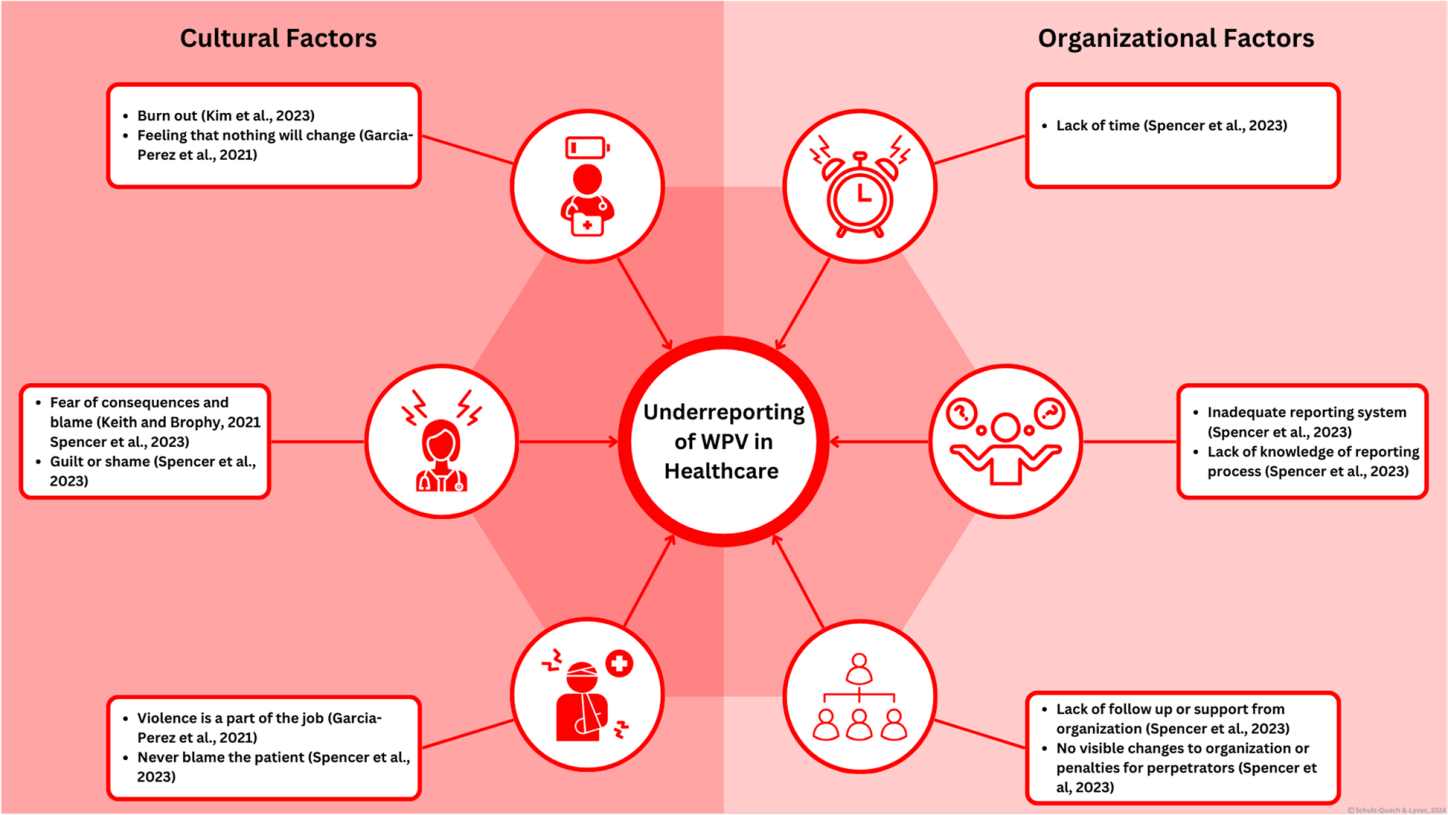
This figure depicts the numerous cultural and organizational factors contributing to the underreporting of WPV in healthcare. References related to [7, 53, 106, 107]

The underreporting of WPV is a culturally and structurally rooted problem faced by healthcare institutions. Research has documented that as many as 88% of HCPs that experienced WPV did not report the event [104]. WPV reporting is crucial to identifying WPV trends and informing decision making processes. A wide array of cultural and organizational factors contributes to underreporting (Fig. 7), many of which can be addressed through an updated WPV reporting system. Staff require a convenient, accessible reporting system that minimizes added workload, provides staff with follow up messages to demonstrate a course of action was taken and provides WPV support resources to ensure that staff feel seen, heard, supported, protected, and cared for [68, 105]. Education interventions and debriefs must encourage the use of WPV reporting systems to create a culture of reporting.

### Step 12: implement comprehensive training plan based on HCP’s environmental risks

Implementing a new or updated training plan that meets the needs of their staff is a crucial step in addressing WPV in healthcare [108]. Effective WPV prevention training improves the management of WPV situations, increases staff’s sense of safety and promotes a culture of safety within the organization [109]. Training must include simulation and education programs that focus on WPV awareness, verbal and physical de-escalation, agitation management, decision making, critical thinking, crisis intervention training and conflict resolution to be effective [7, 110]. Research indicates that factors such as an employee's department, frequency of patient interactions, and concerns regarding WPV are key contributors to the likelihood of their involvement in a WPV incident [111]. Consequently, staff’s training requirements need to be determined by generating risk profiles that consider these factors rather than relying solely on professions as a determining factor of needs. Utilizing risk profiles for training will lead to interdisciplinary cohorts that will enhance staff’s understanding of other roles, interprofessional communication and teamwork [112]. In our case example at our organizations, criteria for risk level stratification were based upon an environmental assessment of each unit.

### Limitations

Although the development of our QI project emphasized the utilization of a systemic, methodological approach, there are several limitations that require acknowledgement. It is important to note that the objective of this paper is to illustrate the development of a systemic WPV QI project, rather than its success. Consequently, this article lacks data to demonstrate the effectiveness of the interventions. Our team is in the process of publishing findings of the individual subprojects.

An additional limitation of the framework is its limited generalizability. The subprojects are specific to our healthcare institution, shaped by resources and processes unique to our situation. For example, not all healthcare institutions utilize the Code White response protocol or manage WPV incidents using physical restraint systems. Furthermore, the timing of this project’s initiation during the COVID-19 pandemic recovery phase may have influenced our findings. The global surge in WPV during this period created a distinctive environment [3, 4], which may limit the applicability of the framework in other healthcare settings. Moreover, sustainability of large-scale QI projects is difficult due to interventions, such as education and training, requiring ongoing funding and key collaborators support.

## Conclusions

In conclusion, WPV is a multifactorial and complex phenomenon in healthcare which ought to be addressed through complex interventions and a systems approach. Through the utilization of the SEIPS 3.0 framework and SEIPS 101 tools, our team developed such an approach to address WPV at a multi-site academic health sciences centre in Toronto, Ontario. In addition, we have developed a framework outlining the necessary steps that we undertook in developing our own project. This framework can be utilized by healthcare institutions to aid in establishing a comprehensive WPV QI project within their own settings. Future research should focus on factors that optimize WPV QI engagement and intervention impact on the organizational level.

## Supplementary Information

Supplementary Material 1.

Supplementary Material 2.

Supplementary Material 3.

Supplementary Material 4.

## Data Availability

All data generated or analyzed during this study are included in this published article [and its supplementary information files].

## Abbreviations

WPV: Workplace violence
ED: Emergency Department
QI: Quality improvement
SPO: Structure-Process-Outcome
HCP: Healthcare Provider
SEIPS: System Engineering Imitative for Patient Safety
PETT: People, Environment, Tools, Tasks
SP: Subproject
DICE: Duration, Integrity, Commitment and Effort
