# Supplementary material for: Understanding and measuring workplace violence in healthcare: a Canadian systematic framework to address a global healthcare phenomenon
Source: BMC Emerg Med. 2025 Jan 13;25:9. doi: 10.1186/s12873-024-01144-1 (PMC11727261; doi:10.1186/s12873-024-01144-1)
Supplement: Supplementary file 1 — Supplementary Material 1. [file 12873_2024_1144_MOESM1_ESM.pdf]

Literature Based Interventions

The first two subprojects of our QI initiative draw from existing literature. We began an ongoing literature review (SP1) of WPV-related topics including: (1) WPV in healthcare, (2) agitation management, (3) de-escalation techniques and (4) Code White simulation training as an educational tool. The findings from these literature reviews assisted in guiding the development of our interventions. 84 articles published from 2012 to 2024 were reviewed. In addition, from the inception of the project, it was evident that a novel approach to measuring and monitoring WPV within our organization was imperative [21]. To address this requirement, our team performed a rapid review to identify 229 quality indicators previously used to measure WPV in healthcare environments (SP2) [8].

Outreach Interventions

Two subprojects placed an emphasis on collecting qualitative feedback and guidance from the parties involved in WPV incidents. This consisted of performing qualitative interviews with ED staff (SP4) at four time points related to the implementation of new interventions. Our first round (December 2022) included 52 interviews with ED staff and the second round (May 2023) included 23 interviews with ED staff. ED staff were selected using a convenience sampling strategy. Further post-implementation interview rounds are scheduled for the future (Figure 3). Additionally, efforts to create a patient partner and community outreach group (SP12) were made to elicit the perspectives of all parties involved in WPV. As a trauma-informed approach was emphasized throughout this project's development, it was pivotal to ensure that patients, caregivers, and chosen family members were included [22]. Additionally, this initiative can serve as an opportunity to repair relationships with patients involved in WPV events as up to 66% of individuals who underwent physical restraint indicated feelings of distrust towards HCPs, emotional trauma, and subsequent delays or omissions in attending future treatment sessions [23]. As of February 2024, four meetings have taken place to orchestrate this initiative.

Educational Interventions

Two additional subprojects addressed our healthcare institution's requirement for WPV training. At the inception of our QI project, training related to WPV was reduced to e-learning modules owing to Covid-19 pandemic restrictions. Once these restrictions were lifted, an ad-hoc training program (SP5, N = 220) was implemented to address urgent requests for verbal de-escalation and situational awareness training in our EDs. We also utilized this project as a proof-of-concept demonstrating the feasibility of organization-wide in-person WPV prevention training. While satisfied with the point-of-care training, staff requested a comprehensive training model that placed emphasis on trauma-informed care, de-escalation, and self-protection skills. Consequently, a second training initiative (SP8) was implemented to create a three-day risk-specific trauma-informed training program. This training is based on the Trauma-Informed De-Escalation Education for Safety and Self-Protection (TIDES) program [24]. An adaptation of this program was developed in partnership with the psychiatric teaching hospital in Toronto, Ontario where TIDES was originally developed. Clinical leaders, organizational WPV

experts, and organizational partners including the Joint Health and Safety Committee contributed to the development and implementation process. Two education specialists were hired, spearheading twelve in-house trainer dyads to deliver the training. Each training session was led by a dyad consisting of a clinician and Security representative to ensure increased interdisciplinary collaboration and role modeling. An organization-wide rollout has begun with an emphasis on high-risk areas at UHN (e.g. EDs, specialized inpatient units).

Organizational Interventions

Several subprojects aimed to address organizational needs at UHN as they pertain to WPV. Once a compilation of WPV quality indicators was assembled, the top-quality indicators for UHN (SP3) were selected through a Delphi process that included key collaborators and experts in WPV [21]. 17 quality indicators were identified and operationalized through a digital WPV dashboard at our organization using data from nine separate organizational databases as our sources.

Additionally, structural changes were required for processes related to WPV and Code White incidents. A Code White Governance Committee (SP6) was established to evaluate and streamline processes involved in Code White incidents. The committee performed an organization-wide needs assessment that included qualitative interviews and focus groups with patient facing staff (n=257). The needs assessment demonstrated that UHN required improved WPV training, standardized response to Code White incidents and designated Code White specialists. In addition, the committee developed the 'Life cycle of a Code White' to determine which processes required updating [2]. Procedures that required updating included WPV and Code White incident reporting (SP7), debriefing (SP10) and physical restraint systems (SP11). Consequently, reviewing, updating, and implementing modifications to these processes evolved into new subprojects. Numerous functional units collaborated to develop an efficient, user-friendly, trauma-informed reporting system with 14 meetings dedicated to achieving this feat. Furthermore, functional units collaborated to implement a hot and cold debrief model and an algorithm for flagging a WPV event as a 'Code White not called'. A hot debrief is a debrief with all staff involved in the event within hours of the event's occurrence [2]. A cold debrief is a more in-depth debrief facilitated by the Code White Governance Committee and Emergency Preparedness that involves all staff involved in the event [2]. Cold debriefs are only necessary when further investigation and resolution is required. Following the implementation of these processes, there have been 304 hot debriefs, 13 cold debriefs and 17 requests to flag a WPV event as a 'Code White not called'. As well, policy changes were implemented at UHN to establish a standardized approach to physical restraint utilization, aligning with the objectives of a new training program and emphasizing the potential impact on patients. Nine meetings were dedicated to the development of physical restraint systems at UHN.

Lastly, an environmental indicator project (SP9) was implemented within UHN's EDs. Five posters were developed to communicate messages of mutual respect between patients and HCPs using trauma-informed language (Figure 4). The research team collected feedback from 106 staff members through surveys. Although staff voiced that the posters were not enough to

prevent WPV, they supported the project and requested that more posters be placed throughout the hospital (Figure 4). In addition, the Department of Security Operations at our organization implemented multiple interventions alongside our QI project, including increasing the number of security guards in the ED from one to two guards at all times, as well as introducing wearable devices (e.g. body cameras) for all security guards. Both interventions were received positively by ED staff according to the findings from our qualitative interviews.
