# Supplementary material for: Understanding and measuring workplace violence in healthcare: a Canadian systematic framework to address a global healthcare phenomenon
Source: BMC Emerg Med. 2025 Jan 13;25:9. doi: 10.1186/s12873-024-01144-1 (PMC11727261; doi:10.1186/s12873-024-01144-1)
Supplement: Supplementary file 3 — Supplementary Material 3. [file 12873_2024_1144_MOESM3_ESM.pdf]

INTERVIEW GUIDE (WORKPLACE-BASED VIOLENCE AND CODE WHITE INCIDENCES)

Before we begin, I would like to provide you with a breakdown of the structure of this interview. We will try to collect information on your sense of safety, sense of support from leadership, your perspective of UHN security and personal expectations of what change you would like to see happen at UHN. We have collected a number of vignettes that we have paired with these topics. The vignettes are from a book called Code White which provides an in-depth summary of the workplace violence issues in Canadian health care. Some of these vignettes include healthcare provider stories that can be quite triggering due to the violence and dangerous situations that healthcare providers have faced in the past. While we feel that it is important to acknowledge and talk about these stories, we understand that this is a very sensitive topic so please let us know if you would like to opt out of this interview before we begin or feel at any point that you need a break or would like to step away from this interview.

To begin, is this the first or second time that I have interviewed you on workplace violence in the emergency department?

(If this is the first interview, go to guide A. If this is the second interview, go to Guide B.)

Guide A

1. Sense of safety

VIGNETTE:

[A senior psychiatric nurse] relieved the nurse who was sitting with [the patient] and found her patient to be on his bed crying. When she asked what was wrong, he said he wanted to use the phone. She calmly explained that he could use it as soon as the other staff returned from their dinner breaks. He began demanding the phone and she repeated that he would have to wait a little while. He quickly turned from unhappy to irate. He jumped up from the bed, yelling something unintelligible, and grabbed a chair. Dianne turned to leave the room but found that the door had closed behind her. Then he unleashed his fury.

"I was barricaded behind the door with the chair hard against me. He pounded me on my head, my face, and on my shoulders. He was yelling and screaming while he punched me. And a patient who was on the other side of the door started yelling and screaming."

1. In these types of scenarios, do you feel that UHN provides you with the tools to manage the situation safely?
2. Working with agitated patients can be unavoidable in certain scenarios, what would help you to feel confident and safe when working with agitated patients?
3. How important is it that you and your colleagues feel safe in your workspace?
4. Which of the following interventions do you think would improve your sense of safety when working with agitated patients?
    a. Implementing environmental indicators for harm reduction and risk minimization
    b. Personal alarm buttons for clinicians and staff
    c. Additional security guard at each emergency department
    d. Wearable video solutions (ie. Body cams) for security guards
    e. Flagging patients with history of violence or complex mental health conditions

1. <u>Sense of support and training provided by leadership</u>

VIGNETTE A:

"He just lost it.

There was two of us in the area at the time. It was hard to keep him calm, even with [de-escalation techniques]...I had a hold of him at one time-and I remember him throwing furniture....

And then some other staff came in. We got him to the ground but only after my co-worker almost went through the glass window...The staff called a Code White, but the team couldn't get on the unit. The doors had malfunctioned...Nobody had a key that could work. So it was just us – the staff on the floor trying to contain the other patients and the patient who was acting out."

VIGNETTE B:

Many of the healthcare workers talked about the hurt and disrespect they felt because of their management's apparent lack of sympathy or concern after a violent incident. One participant emphasized the need for those in positions of authority to be trained in "compassion."

1. Do you feel that UHN provides you with the education, training and support necessary to handle these types of scenarios?
2. What type of training or support would you expect leadership to provide for you to feel confident in de-escalating situations such as this?
3. Which of the following interventions would improve your sense of support and training provided by leadership?
    a. Implementing de-escalation training consisting of both verbal and non-verbal de-escalation techniques
    b. Implementing Code White simulation training
    c. Implementing a dedicated UHN Code White Governance Committee
    d. Reviewing, updating, and implementing incident reporting for WPV and Code White incidents
    e. Implementing check-ins with clinicians and staff to collect feedback on perceptions of safety, support, and clinical guidance relating to managing and learning from WPV incidences

2. Perspective of Security at UHN

VIGNETTE:

Many of the staff we talked to said that security is lacking or insufficient in the facilities they work in. They believe they would be better protected if there were an increase in the number of security personnel with higher levels of training. In some cases, they should be equipped to intervene with violent individuals.

1. What is your initial thought when you see a UHN security guard?
2. What are your current perspectives on UHN security guards?
3. In what way do you think security guards are part of the medical team?
4. Do you feel that the security guards work with you to improve patient experience and safety?
5. Do UHN Security guards contribute to your own personal sense of safety?

3. Personal expectations of what needs to change

VIGNETTE A:

In 2018, an ER nurse…was brutally assaulted by a patient. The attack left her with long-lasting psychological injuries, such as frightening flashbacks, panic attacks, and depression. She told CBC News:

"It's absolutely awful. I tried to go to counselling. I'm on anti-depressants now. I feel totally defeated. Just defeated. Because nobody's really doing anything about it. Every single day there's a violent incident in HSC emerge. Every day. And no one seems to acknowledge it"

So the question emerges: Can anything really be done to protect healthcare staff from violence?

VIGNETTE B:

How might healthcare workers overcome…daunting hurdles and achieve the protections, respect, support, and rights that they deserve? This, we believe will require the energy and power of a broadly based social movement. As an exasperated hospital clerical worker told us:

"There comes a time when you've got to say, "Enough is enough." I think this is what we're trying to do now, to fight back…We need to get the message out."

1. How does this passage resonate with you?
2. What are your thoughts on what can be done to protect healthcare staff from violence based on your personal experience?

UHN Security
PREVENTION • LEADERSHIP • RESPONSE

Guide B

1. Since I last spoke to you, what is your current experience of workplace violence in the emergency department?

2. What are your experiences with verbal harassment and threats towards you or your colleagues in the emergency department?

3. What are your experiences of incidents of harm involving bodily fluids towards you or your colleagues in the emergency department?

4. Have you experienced psychological trauma following a workplace violence incident in the emergency department?

5. Could you describe any workplace violence incidents that you would consider preventable?

6. How would you evaluate staff's response time to code white events?

7. Have you been offered support by UHN following a workplace violence incident in the emergency department? If so, were they helpful? If not, what types of support should be available?

8. What is your perception of organizational support from UHN?

9. Have you noticed any differences in your interactions with the UHN Security Team?

10. How would you describe your current feelings of well-being and safety when working in the emergency department?

11. What are your thoughts on what can be done to protect healthcare staff from violence based on your personal experience?

References

1. Keith, M. M., & Brophy, J. T. (2021). *Code White*. Between the Lines.
