# Supplementary material for: Understanding and measuring workplace violence in healthcare: a Canadian systematic framework to address a global healthcare phenomenon
Source: BMC Emerg Med. 2025 Jan 13;25:9. doi: 10.1186/s12873-024-01144-1 (PMC11727261; doi:10.1186/s12873-024-01144-1)
Supplement: Supplementary file 4 — Supplementary Material 4. [file 12873_2024_1144_MOESM4_ESM.pdf]

UHN Security

PREVENTION • LEADERSHIP • RESPONSE

UHN Security QI Project: Environmental Indicators

Mutual Respect

Dear Esteemed Colleague,

We would like to thank you for engaging in our quality improvement project for the University Health Network (UHN). Currently, a multi-level quality improvement project is underway, addressing workplace violence (WPV) and Code White incident management across all sites at UHN. The focus of this subproject (project 9 of 12) is on environmental indicators for harm reduction and risk minimization.

At the end of March, 5 posters went live on all Emergency Department Waiting Room screens for the first digital pilot phase of the project. This was a concerted effort between the UHN Workplace Violence Advisory Board, UHN Security, UHN Safety Services, UHN Emergency Preparedness and the UHN Workplace Violence Education Collaboration.

We would appreciate if you could please complete this anonymous 2-minute survey to gain your perspective on the workplace violence prevention posters. Your feedback will be used to contribute to adjustments being made to these posters and the decision-making process for a UHN-wide roll out.

-UHN Security QI Team

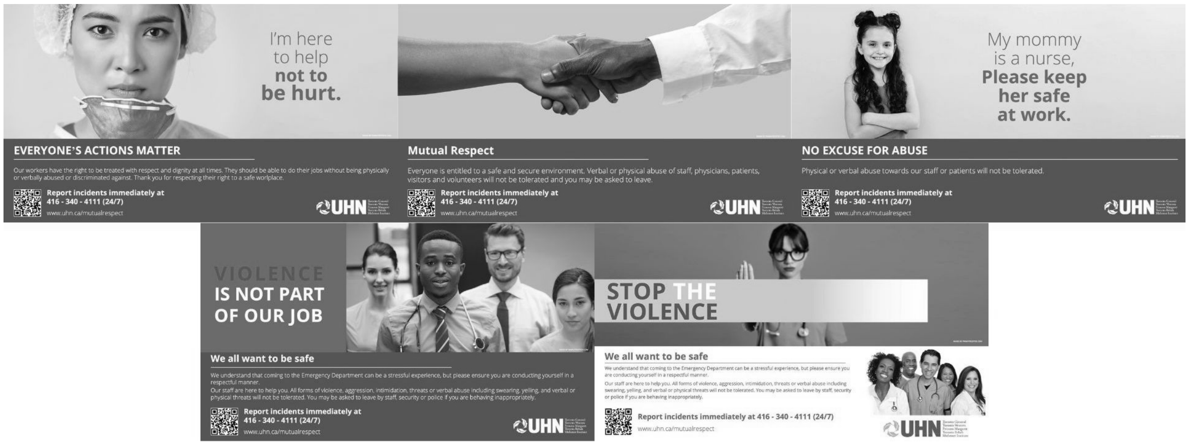

UHN Security
PREVENTION • LEADERSHIP • RESPONSE

UHN Security QI Project: Environmental Indicators

Consent

Information Confidentiality and Dissemination

Taking part in this survey is optional. If you decide not to participate, your employment will not be affected in any way. The information you provide will only be seen by the UHN Security QI Team: two staff physicians, a security director, a project manager and a research analyst. Others within UHN and outside of UHN will only see a summary of the summary data collected. Your responses will not be linked to your name or personal information in any way and will be stored separately from your personal information. It will be kept on a secure UHN server for a two-year period. If the results of this survey interview are published or presented at meetings, your name and other personal identifying information will not be used, and your responses will not be linked to your name or personal information in any way.

Contacts Information

If you have questions about this QI project, please contact Christian Schulz-Quach (Christian.schulz-quach@uhn.ca). If you have questions about your rights as a participant in a UHN Quality Improvement Project, please contact the UHN Quality Improvement Review Committee (QIRC) at QI@uhn.ca. QIRC is a group of people who oversee the ethical conduct of QI projects; they are not part of the project team.

Consent for Future Contact

We are asking for your name and email address to contact you in the future for assessment of the educational materials we develop. If you give us permission to contact you, please fill in your contact information in the space provided. This information will be kept separately from all other information you provide. It will be seen by the UHN Security QI Team and kept in an electronic database for a two-year period.

Thank you for your consideration for participation!

1. Name:

2. Best accessible email:

UHN Security QI Project: Environmental Indicators

Mutual Respect

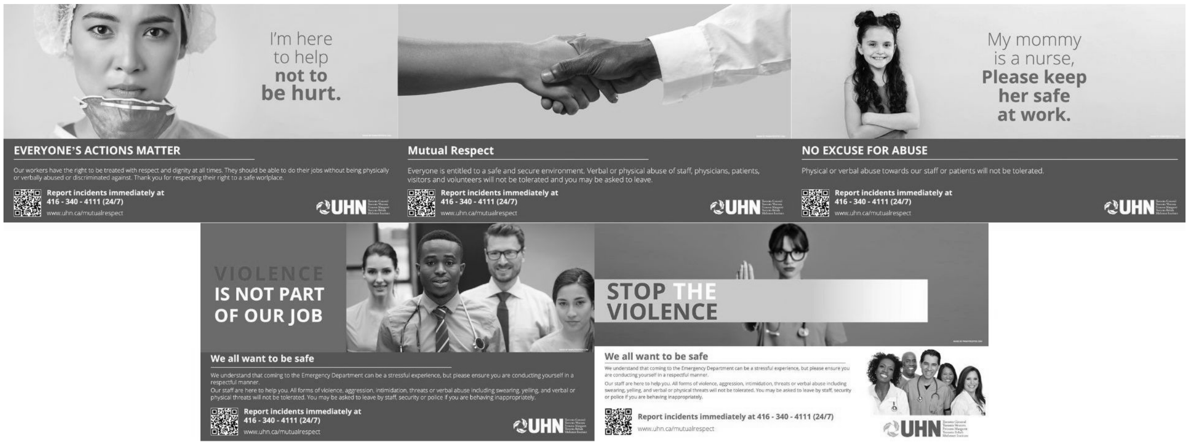

3. Evaluate the following statements.

	Strongly Disagree	Disagree	Neither Agree Nor Disagree	Agree	Strongly Agree
The WPV prevention posters effectively communicate UHN's zero-tolerance policy of WPV to patients.	○	○	○	○	○
Seeing the WPV prevention posters makes me feel supported by UHN.	○	○	○	○	○
Seeing the WPV prevention posters makes me feel safer in my workspace.	○	○	○	○	○

4. The WPV prevention posters should be placed in more locations across the emergency departments.

Strongly Disagree	Disagree	Neither Agree Nor Disagree	Agree	Strongly Agree
○	○	○	○	○

5. Do you have any suggested changes/feedback related to the workplace violence prevention posters?
